# Pseudotumoral Calcinosis Following Bone Fracture

**DOI:** 10.5334/jbsr.1869

**Published:** 2019-10-02

**Authors:** Arnaud Toubeau, Sokol Malasi, Nicolae Sarbu

**Affiliations:** 1 CHU Ambroise Paré, BE; 2 Clinique Sainte-Anne Saint-Rémi, BE; 3 Erasme Hospital, ULB, BE

**Keywords:** Pseudotumoral/tumoral calcinosis, Haemodialysis, MRI, CT

## Abstract

Pseudotumoral calcinosis could present as large heterogeneous calcified masses with fluid levels and sedimentation.

## Case

**Figure 1:**
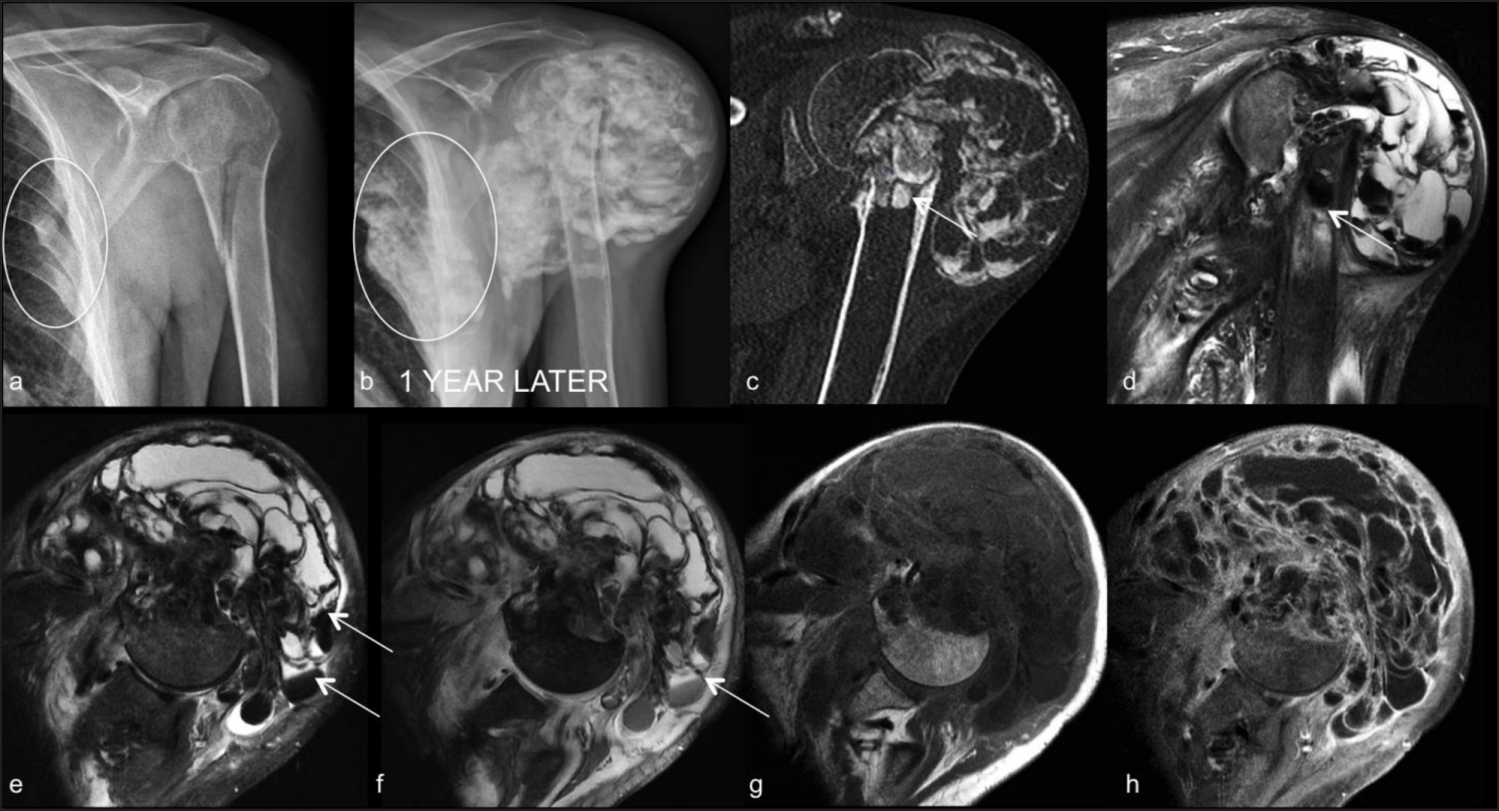
Plain film shows a proximal humeral fracture and rib fractures (circle in **1a**). Plain film 1-year later shows a giant mass involving the left shoulder and a smaller one on the costal grill (circle), both centred on the previous fracture sites (**1b**). CT exam, coronal reconstruction, reveals multiple calcifications within the mass and an extension into the humeral diaphysis (arrow in **1c**). MRI exam (coronal T2-WI Fat Sat in **1d**; axial T2-WI Fat Sat in **1e**; axial GRE sequence in **1f**; axial T1-WI pre- and post- gadolinium administration in **1g, h**) shows the proximal humeral endomedular extension (arrow in 1d) and depicts multiple fluid-calcium levels within the cystic spaces of the lesion (sedimentation sign, arrows in 1d–f), better demonstrating the heterogeneity and the extension of the mass. Contrast-enhanced T1-WI (1h) shows thin peripheral enhancement of the cystic spaces and rules out solid or suspicious components. Abbreviations: CT: computed tomography, MRI: magnetic resonance imaging, T1- and T2-WI: T1- and T2-weighted imaging, GRE: gradient recalled echo.

A 51-year-old male referred to the orthopedist for a left shoulder mass, which had occurred slowly over several months following a fracture of the humeral neck (Figure 1a). Physical exam revealed a lump of the left shoulder with restricted range of motions and loss of strength. The patient was on long-term hemodialysis following nephrectomy for previous malignancy. Plain radiographs and computed tomography (CT) showed a giant calcified and multiloculated mass surrounding the left shoulder, suggesting the diagnosis of pseudotumoral calcinosis. A second similar lesion, along left rib fractures, was also observed (Figure 1a, circles). Magnetic resonance imaging (MRI) showed the polycystic architecture of the mass with fluid levels and sedimentation (Figure 1a, arrows). An in-bone extension of the lesion was found in the proximal diaphysis of the left humerus. The shoulder mass was surgically removed, and histopathology showed hydroxyapatite crystals and synovitis-like findings. No recurrence was present over a span of more than one-year follow-up.

## Comment

Pseudotumoral calcinosis (PTC), sometimes termed tumoral calcinosis, is a rare clinical and histopathologic syndrome, characterized by benign periarticular soft tissue masses. The most frequently involved joints are hip and shoulder, followed by elbow, foot, and wrist. PTC is due to phosphate crystal deposition near medium-to-large articulations, but the underlying pathogenic mechanisms still remain unclear. PTC is classified as primary and secondary. The primary form was mainly reported in patients of African origin typically presenting with large periarticular masses in the first decades of life, and is caused by a hereditary metabolic dysfunction of phosphate regulation associated with massive periarticular calcinosis. The secondary form was frequently associated with secondary hyperparathyroidism in the context of chronic renal failure and haemodialysis. The calcium-phosphorus product is increased in both forms. The mass is usually painless but with functional limitation depending on the location. CT typically shows an expansive mass, adjacent to joints, of cystic appearance, with multiple septa, calcium deposits, and sedimentation. Sedimentation in PTC consists in fluid-calcium levels related to calcium layering within the cystic spaces of the mass. Typically, the underlying bone is normal, without osseous destruction, bone remodelling, or erosions, which is a distinguishing feature of PTC from other pathologies. The role of MRI is to assess the mass extension, assess the relation with local structures, and rule out alternative diagnoses, including myositis ossificans, milk-alkali syndrome, hypervitaminosis D, other forms of calcinosis, synovial osteochondromatosis, gout, and even sarcomas.

The final diagnosis is however often confirmed by histopathology. The atypical intraosseous component of the lesion in our case could be explained by the development and extension of the PTC into the previous fracture line. The treatment frequently involves mass resection while parathyroidectomy remains controversial [1].
